# Supplementary material for: An individualized functional magnetic resonance imaging protocol to assess semantic congruency effects on episodic memory in an aging multilingual population
Source: Front Aging Neurosci. 2022 Jul 22;14:873376. doi: 10.3389/fnagi.2022.873376 (PMC9354990; doi:10.3389/fnagi.2022.873376)
Supplement: Supplementary file 1 [file Data_Sheet_1.PDF]

An individualized fMRI protocol to assess semantic congruency effects on episodic memory in an ageing multilingual population

Magali Perquin, Shivakumar Viswanathan, Michel Vaillant, Okka Risius,
Laetitia Huiart, Jean-Claude Schmit, Nico J. Diederich,
Gereon R. Fink, Juraj Kukolja

Supplementary materials

Table s1. Stimulus materials: words used in English

The Memolingua study protocol used 256 words describing common natural and artificial objects. The colored cells correspond to the words used during encoding, and represent also the position (randomly generated) of the correct answer during picture-word recognition.

N	Stimulus Category	Congruence	Image Name	Word at Encoding	Words at Picture-word recognition			
					Word 1	Word 2	Word 3	Word 4
1	Old	*Unrelated*	I01	rabbit	rabbit	rat	squirrel	dog
2	Old	*Unrelated*	I02	cardigan	sweatshirt	cardigan	clothes	coat
3	Old	*Unrelated*	I03	hen	goose	hen	duck	cock
4	Old	*Unrelated*	I04	nail	hammer	drill	nail	screw
5	Old	*Unrelated*	I05	arrow	crossbow	arrow	bow	target
6	Old	*Unrelated*	I06	spoon	fork	table	plate	spoon
7	Old	*Unrelated*	I07	candy	caramel	chocolate	candy	licorice
8	Old	*Unrelated*	I08	magician	fairy	magician	witch	stick
9	Old	*Unrelated*	I09	fingernail	finger	arm	fingernail	hand
10	Old	*Unrelated*	I10	hair	hair	bun	beard	mustache
11	Old	*Unrelated*	I11	chalet	chalet	hut	house	building
12	Old	*Unrelated*	I12	window	window	shutter	curtain	balcony
13	Old	*Unrelated*	I13	coffee	tea	drink	beer	coffee
14	Old	*Unrelated*	I14	rake	shovel	garden	pickaxe	rake
15	Old	*Unrelated*	I15	rock	rock	pebble	stone	mountain
16	Old	*Unrelated*	I16	book	text	word	newspaper	book
17	Old	*Unrelated*	I17	tooth	bone	skeleton	tooth	jaw
18	Old	*Unrelated*	I18	bread	bread	butter	jam	croissant
19	Old	*Unrelated*	I19	star	star	sky	night	moon
20	Old	*Unrelated*	I20	swallow	zoo	swallow	cage	bird
21	Old	*Unrelated*	I21	walnut	almond	pistachio	walnut	hazelnut
22	Old	*Unrelated*	I22	palm	oak	maple	palm	fir
23	Old	*Unrelated*	I23	soap	shower	bath	towel	soap
24	Old	*Unrelated*	I24	cupboard	dresser	cupboard	shelf	wardrobe
25	Old	*Unrelated*	I25	carafe	bucket	carafe	bottle	basin
26	Old	*Unrelated*	I26	train	bicycle	motorcycle	airplane	train
27	Old	*Unrelated*	I27	gun	sword	saber	carbine	gun
28	Old	*Unrelated*	I28	lion	giraffe	lion	tiger	monkey
29	Old	*Unrelated*	I29	shop	grocery	bakery	shop	butchery
30	Old	*Unrelated*	I30	stairs	elevator	floor	ladder	stairs
31	Old	*Unrelated*	I31	cathedral	mosque	temple	cathedral	synagogue
32	Old	*Unrelated*	I32	heel	heel	shoe	sock	slipper
33	Old	*Related*	I33	water	umbrella	water	rain	mud
34	Old	*Related*	I34	wool	glove	ice	snow	wool
35	Old	*Related*	I35	key	door	lock	safe	key
36	Old	*Related*	I36	suit	shirt	suit	jacket	trousers
37	Old	*Related*	I37	desk	eraser	desk	phone	stapler
38	Old	*Related*	I38	wine	glass	vine	cellar	wine
39	Old	*Related*	I39	goal	match	team	goal	stadium

40	Old	*Related*	I40	sailor	sailor	compass	anchor	lifebelt
41	Old	*Related*	I41	mandarin	grapefruit	mandarin	orange	juice
42	Old	*Related*	I42	stool	sofa	bed	stool	armchair
43	Old	*Related*	I43	nurse	hospital	doctor	bacterium	nurse
44	Old	*Related*	I44	postman	postman	letter	stamp	mail
45	Old	*Related*	I45	net	pond	aquarium	net	algae
46	Old	*Related*	I46	drawing	pencil	drawing	school	color
47	Old	*Related*	I47	ring	ring	woman	jewelry	necklace
48	Old	*Related*	I48	king	poker	ace	king	heart
49	Old	*Related*	I49	crane	crane	facade	tool	scaffolding
50	Old	*Related*	I50	soup	soup	gravy	forest	recipe
51	Old	*Related*	I51	face	eye	nose	face	cheek
52	Old	*Related*	I52	pirate	pirate	hook	treasure	flag
53	Old	*Related*	I53	farm	fence	obstacle	saddle	farm
54	Old	*Related*	I54	hive	swarm	bee	sting	hive
55	Old	*Related*	I55	ashes	firefighter	ashes	fire	alarm
56	Old	*Related*	I56	sea	ocean	sea	beach	sand
57	Old	*Related*	I57	kitchen	meat	kitchen	chef	restaurant
58	Old	*Related*	I58	game	game	six	billiards	pawn
59	Old	*Related*	I59	road	sidewalk	crossing	road	car
60	Old	*Related*	I60	bouquet	rose	vase	plant	bouquet
61	Old	*Related*	I61	orchestra	orchestra	piano	guitar	violin
62	Old	*Related*	I62	sugar	dessert	biscuit	candle	sugar
63	Old	*Related*	I63	clown	acrobat	makeup	clown	circus
64	Old	*Related*	I64	child	girl	boy	child	baby
65	New	not applicable	I65					
66	New	not applicable	I66					
67	New	not applicable	I67					
68	New	not applicable	I68					
69	New	not applicable	I69					
70	New	not applicable	I70					
71	New	not applicable	I71					
72	New	not applicable	I72					
73	New	not applicable	I73					
74	New	not applicable	I74					
75	New	not applicable	I75					
76	New	not applicable	I76					
77	New	not applicable	I77					
78	New	not applicable	I78					
79	New	not applicable	I79					
80	New	not applicable	I80					
81	New	not applicable	I81					
82	New	not applicable	I82					
83	New	not applicable	I83					
84	New	not applicable	I84					
85	New	not applicable	I85					
86	New	not applicable	I86					

87	New	not applicable	I87					
88	New	not applicable	I88					
89	New	not applicable	I89					
90	New	not applicable	I90					
91	New	not applicable	I91					
92	New	not applicable	I92					
93	New	not applicable	I93					
94	New	not applicable	I94					
95	New	not applicable	I95					
96	New	not applicable	I96					

Table s2. Stimulus materials: words used in French

The Memolingua study protocol used 256 words describing common natural and artificial objects.
The colored cells correspond to the words used during encoding, and represent also the position (randomly generated) of the correct answer during picture-word recognition.

Image Name	Word at Encoding	Words at Picture-word recognition			
		Word 1	Word 2	Word 3	Word 4
I01	lapin	lapin	rat	écureuil	chien
I02	gilet	pull	gilet	habit	manteau
I03	poule	oie	poule	canard	coq
I04	clou	marteau	perceuse	clou	vis
I05	flèche	arbalète	flèche	arc	cible
I06	cuillère	fourchette	table	assiette	cuillère
I07	bonbon	caramel	chocolat	bonbon	réglisse
I08	magicien	fée	magicien	sorcière	baguette
I09	ongle	doigt	bras	ongle	main
I10	cheveux	cheveux	chignon	barbe	moustache
I11	chalet	chalet	cabane	maison	immeuble
I12	fenêtre	fenêtre	volets	rideaux	balcon
I13	café	thé	boisson	bière	café
I14	râteau	pelle	jardin	pioche	râteau
I15	rocher	rocher	caillou	pierre	montagne
I16	livre	texte	mot	journal	livre
I17	dent	os	squelette	dent	mâchoire
I18	pain	pain	beurre	confiture	croissant
I19	étoile	étoile	ciel	nuit	lune
I20	hirondelle	zoo	hirondelle	cage	oiseau
I21	noix	amende	pistache	noix	noisette
I22	palmier	chêne	érable	palmier	sapin
I23	savon	douche	bain	serviette	savon
I24	placard	commode	placard	étagère	armoire
I25	carafe	sceau	carafe	bouteille	bassine
I26	train	vélo	moto	avion	train
I27	pistolet	épée	sabre	carabine	pistolet

I28	lion	girafe	lion	tigre	singe
I29	magasin	épicerie	boulangerie	magasin	boucherie
I30	escaliers	ascenseur	étage	échelle	escaliers
I31	cathédrale	mosquée	temple	cathédrale	synagogue
I32	talon	talon	chaussures	chaussette	pantoufle
I33	eau	parapluie	eau	pluie	boue
I34	laine	gant	glace	neige	laine
I35	clé	porte	serrure	coffre	clé
I36	costume	chemise	costume	veste	pantalon
I37	bureau	gomme	bureau	téléphone	agrafeuse
I38	vin	verre	vigne	cave	vin
I39	but	match	équipe	but	stade
I40	marin	marin	boussole	ancre	bouée
I41	mandarine	pamplemousse	mandarine	orange	jus
I42	tabouret	canapé	lit	tabouret	fauteuil
I43	infirmière	hôpital	docteur	bactérie	infirmière
I44	facteur	facteur	lettre	timbre	courrier
I45	filet	étang	aquarium	filet	algue
I46	dessin	crayon	dessin	école	couleur
I47	bague	bague	femme	bijou	collier
I48	roi	poker	as	roi	cœur
I49	grue	grue	façade	outil	échafaudage
I50	soupe	soupe	sauce	forêt	recette
I51	visage	œil	nez	visage	joue
I52	pirate	pirate	perroquet	trésor	drapeau
I53	ferme	clôture	obstacle	selle	ferme
I54	ruche	essaim	abeille	piqure	ruche
I55	cendres	pompier	cendres	feu	alarme
I56	mer	océan	mer	plage	sable
I57	cuisine	viande	cuisine	chef	restaurant
I58	jeu	jeu	six	billard	pion
I59	route	trottoir	croisement	route	voiture
I60	bouquet	rose	vase	plante	bouquet
I61	orchestre	orchestre	piano	guitare	violon
I62	sucre	dessert	biscuit	bougie	sucre
I63	clown	acrobate	maquillage	clown	cirque
I64	enfant	fille	garçon	enfant	bébé

Table s3. Stimulus materials: words used in German

The Memolingua study protocol used 256 words describing common natural and artificial objects. The colored cells correspond to the words used during encoding, and represent also the position (randomly generated) of the correct answer during picture-word recognition.

Image Name	Word at Encoding	Words at Picture-word recognition			
		Word 1	Word 2	Word 3	Word 4
I01	Hase	Hase	Ratte	Eichhörnchen	Hund

I02	Strickjacke	Pullover	Strickjacke	Kleidung	Mantel
I03	Henne	Gans	Henne	Ente	Hahn
I04	Nagel	Hammer	Bohrer	Nagel	Schraube
I05	Pfeil	Armbrust	Pfeil	Bogen	Zielscheibe
I06	Löffel	Gabel	Tisch	Teller	Löffel
I07	Süßigkeiten	Karamell	Schokolade	Süßigkeiten	Lakritz
I08	Zauberer	Fee	Zauberer	Hexe	Zauberstab
I09	Fingernagel	Finger	Arm	Fingernagel	Hand
I10	Haare	Haare	Dutt	Bart	Schnurrbart
I11	Hütte	Hütte	Bude	Haus	Gebäude
I12	Fenster	Fenster	Rolladen	Vorhang	Balkon
I13	Kaffee	Tee	Getränk	Bier	Kaffee
I14	Rechen	Schaufel	Garten	Spitzhacke	Rechen
I15	Felsen	Felsen	Kiesel	Stein	Berg
I16	Buch	Text	Wort	Zeitung	Buch
I17	Zahn	Knochen	Skelett	Zahn	Kiefer
I18	Brot	Brot	Butter	Marmelade	Croissant
I19	Stern	Stern	Himmel	Nacht	Mond
I20	Schwalbe	Zoo	Schwalbe	Käfig	Vogel
I21	Walnuss	Mandel	Pistazie	Walnuss	Haselnuss
I22	Palme	Eiche	Ahorn	Palme	Tanne
I23	Seife	Dusche	Bad	Handtuch	Seife
I24	Wandschrank	Kommode	Wandschrank	Regal	Kleiderschrank
I25	Karaffe	Eimer	Karaffe	Flasche	Schale
I26	Zug	Fahrrad	Motorrad	Flugzeug	Zug
I27	Pistole	Schwert	Säbel	Jagdgewehr	Pistole
I28	Löwe	Giraffe	Löwe	Tiger	Affe
I29	Laden	Lebensmittellade	Bäcker	Laden	Fleischerei
I30	Treppen	Fahrstuhl	Etage	Leiter	Treppen
I31	Kathedrale	Moschee	Tempel	Kathedrale	Synagoge
I32	Absatz	Absatz	Schuh	Socke	Pantoffel
I33	Wasser	Schirm	Wasser	Regen	Schlamm
I34	Wolle	Handschuh	Eis	Schnee	Wolle
I35	Schlüssel	Tür	Schlüsselloch	Tresor	Schlüssel
I36	Anzug	Hemd	Anzug	Jacke	Hose
I37	Schreibtisch	Radiergummi	Schreibtisch	Telefon	Tacker
I38	Wein	Glas	Rebe	Keller	Wein
I39	Tor	Spiel	Mannschaft	Tor	Stadion
I40	Matrose	Matrose	Kompass	Anker	Rettungsring
I41	Mandarine	Pampelmuse	Mandarine	Orange	Saft
I42	Hocker	Sofa	Bett	Hocker	Sessel
I43	Krankenschwester	Krankenhaus	Arzt	Bakterien	Krankenschwester
I44	Postbote	Postbote	Brief	Briefmarke	Post
I45	Netz	Teich	Aquarium	Netz	Alge
I46	Zeichnung	Stift	Zeichnung	Schule	Farbe
I47	Ring	Ring	Frau	Schmuck	Kette
I48	König	Poker	Ass	König	Herz

I49	Kran	Kran	Fassade	Werkzeug	Gerüst
I50	Suppe	Suppe	Soße	Wald	Rezept
I51	Gesicht	Auge	Nase	Gesicht	Wange
I52	Pirat	Pirat	Papagei	Schatz	Flagge
I53	Bauernhof	Zaun	Hürde	Sattel	Bauernhof
I54	Bienenstock	Schwarm	Biene	Stich	Bienenstock
I55	Asche	Feuerwehrmann	Asche	Feuer	Alarm
I56	Meer	Ozean	Meer	Strand	Sand
I57	Küche	Fleisch	Küche	Chefkoch	Restaurant
I58	Spiel	Spiel	Sechs	billiard	Spielfigur
I59	Straße	Bürgersteig	Kreuzung	Straße	Auto
I60	Strauß	Rose	Vase	Pflanze	Strauß
I61	Orchester	Orchester	Klavier	Gitarre	Geige
I62	Zucker	Nachtisch	Keks	Kerze	Zucker
I63	Clown	Akrobat	Schminke	Clown	Zirkus
I64	Kind	Mädchen	Junge	Kind	Baby

Table s4. Stimulus materials: words prepared in Luxembourgish (but not used)

The Memolingua study protocol used 256 words describing common natural and artificial objects. The colored cells correspond to the words used during encoding, and represent also the position (randomly generated) of the correct answer during picture-word recognition.

Image Name	Word at Encoding	Words at Picture-word recognition			
		Word 1	Word 2	Word 3	Word 4
I01	Hues	Hues	Raat	Kaweechelchen	Hond
I02	Gilet	Pullover	Gilet	Kleeder	Mantel
I03	Hong	Gäns	Hong	Int	Hunn
I04	Nol	Hummer	Buermaschinn	Nol	Schrauf
I05	Feil	Armbrust	Feil	Bou	Zilscheif
I06	Läffel	Forschett	Desch	Teller	Läffel
I07	Bonbon	Kramel	Schockela	Bonbon	Mokuch
I08	Zauberer	Fee	Zauberer	Hex	Stäbercher
I09	Krall	Fanger	Aarm	Krall	Hand
I10	Hoer	Hoer	Chignon	Baart	Schnauz
I11	Chalet	Chalet	Bud	Haus	Gebai
I12	Fenster	Fenster	Lueden	Rido	Balkon
I13	Kaffi	Tei	Gedrenks	Béier	Kaffi
I14	Reech	Schepp	Gaart	Pioche	Reech
I15	Fiels	Fiels	Wak	Steen	Bierg
I16	Buch	Text	Wuert	Zeitung	Buch
I17	Zant	Knachen	Skelett	Zant	Gebéck
I18	Brout	Brout	Botter	Gebees	Croissant
I19	Star	Star	Himmel	Nuecht	Mound
I20	Schmuebel	Zoo	Schmuebel	Käfeg	Villchen
I21	Noss	Mandel	Pistache	Noss	Hieselnoss
I22	Palm	Eech	Ahorn	Palm	Dänn

I23	Seef	Dusch	Bued	Duch	Seef
I24	Wandschaaf	Kammoud	Wandschaf	Bicherregal	Kleederschaf
I25	Karaff	Eemer	Karaff	Fläsch	Baséng
I26	Zuch	Velo	Moto	Fliger	Zuch
I27	Pistoul	Schwert	Säbel	Flent	Pistoul
I28	Léiw	Giraff	Léiw	Tiger	Af
I29	Buttek	Epicerie	Bäckerei	Buttek	Metzlerei
I30	Trapenhaus	Lift	Stack	Leeder	Trapenhaus
I31	Kathedral	Moschee	Tempel	Kathedral	Synagog
I32	Tallek	Tallek	Schong	Strëmp	Schlapp
I33	Waasser	Prabbeli	Waasser	Reen	Bulli
I34	Woll	Händsch	Äis	Schnéi	Woll
I35	Schlëssel	Dier	Schlass	Kofferfort	Schlëssel
I36	Kostüm	Hiem	Kostüm	Paltong	Box
I37	Büro	Gummi	Büro	Telefon	Agrafeuse
I38	Wäin	Glas	Drauw	Keller	Wäin
I39	Goal	Partie	Equipe	Goal	Stadion
I40	Matrous	Matrous	Kompass	Anker	Rettungsrank
I41	Mandarin	Pampelmousse	Mandarin	Orange	Jus
I42	Hocker	Couche	Bett	Hocker	Fotell
I43	Krankeschwëster	Spidol	Dokter	Microbe	Krankeschwëster
I44	Bréifdréier	Bréifdréier	Bréiw	Timber	Post
I45	Netz	Weier	Aquarium	Netz	Alg
I46	Zeechnung	Bläistëft	Zeechnung	Schoul	Faarf
I47	Rank	Rank	Fra	Schmuck	Ketten
I48	Kinnek	Pouker	Ass	Kinnek	Häerz
I49	Kran	Kran	Fassad	Handwierksgeschir	Stee
I50	Zopp	Zopp	Zooss	Bësch	Rezept
I51	Gesiicht	Aa	Nues	Gesiicht	Bak
I52	Pirat	Pirat	Papagei	Schatz	Fändel
I53	Bauerenhaff	Zonk	Hindernis	Suedel	Bauerenhaff
I54	Stack	Schwarm	Bei	Stach	Beiestack
I55	Aeschen	Pompjee	Äschen	Feier	Alarm
I56	Mier	Ozean	Mier	Strand	Sand
I57	Kichen	Fleesch	Kichen	Chef	Restaurant
I58	Spill	Spill	Sechs	Billard	Deppchen
I59	Strooss	Greng	Kräizung	Strooss	Auto
I60	Bouquet	Rous	Vas	Planz	Bouquet
I61	Orchester	Orchester	Piano	Gitar	Gei
I62	Zocker	Dessert	Kichelchen	Käerz	Zocker
I63	Clown	Akrobat	Schminke	Clown	Zirkus
I64	Kand	Meedchen	Jong	Kand	Puppelchen

Table s5. Pictures selected from normed databases and used in the study (n=96)

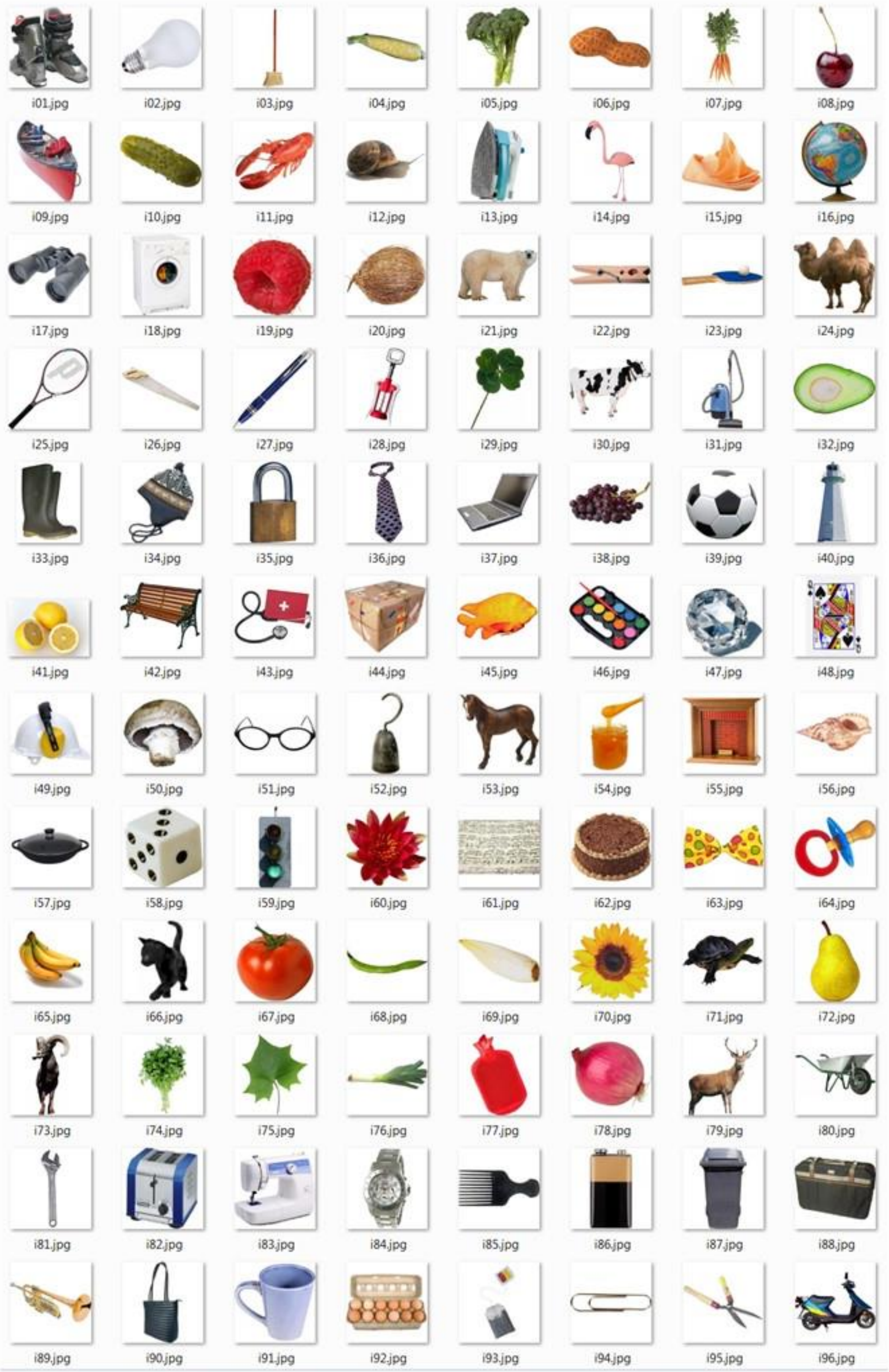

Table s6. Summary of sample sizes in the different analyses.

Missing data are indicated by gray cells.

	Sample sizes				
**Analysis**	**GER**	**FR**	**ENG**	**LUX**	**Total**
**Language choice**	42	18	2	0	62
**Incomplete data**	1	1	0	0	2
**Encoding**					
Accuracy	42	18	2	0	62
RT	42	18	2	0	62
fMRI	41	17	2	0	60
**Picture recogn.**					
Accuracy	42	18	2	0	62
RT	42	18	2	0	62
fMRI	41	17	2	0	60
**Picture-word recogn.**					
Accuracy	42	18	2	0	62
RT	41	17	2	0	60
fMRI	41	17	2	0	60
**Language subgroup comparison**					
Behavior	42 (41)	18 (17)	-	-	
fMRI	41	17	-	-	

Table s7. Comparison between standard and trimmed mean RTs for the three fMRI sessions.

Trimmed means are shown in the left columns and the standard (non-trimmed) means are show in the right columns. For each comparison, the population data were first assessed for normality (using the Kolmogorov-Smirnov test). The *t*-test was used for normal data, and the non-parametric Wilcoxon signed-rank test (*Z* statistic) was used for non-normal data. Note that in two scenarios (Encoding: *Related* vs *Unrelated*, and Picture-recognition: *Related Old* vs *Unrelated Old*), the trimmed estimates were not normally distributed while the corresponding non-trimmed estimates were normally distributed. Therefore, for comparability, we additionally report the non-parametric statistic (in light gray font) along with the allowed parametric statistic. The values in red show the mean difference in RT. Inter-condition RT differences that were statistically significant with trimmed means remained so with the standard means and differences that were not significant also remained unchanged.

Encoding:

Response time: **Trimmed means**			Response time: **Means**		
Related	Unrelated	statistic	Related	Unrelated	statistic
1749 ± 452 ms	1877 ± 431 ms<br>*+7.3%*	Z[61]=657.5<br>p < 0.0001	1872 ± 418 ms	1957 ± 421 ms<br>*+4.5%*	t[61]=3.42<br>p = 0.0011<br>Z[61]=494.5<br>p < 0.0003

Picture recognition:

Response time: **Trimmed means**				Response time: **Means**			
Related Old	Unrel. Old	New	statistic	Related Old	Unrel. Old	New	statistic
1082 ± 158ms	1247 ± 236ms<br>*+15.2%*	-	Z[61]=905.5<br>p < 0.0001	1157 ± 172ms	1354 ± 267ms<br>*+17.0%*	-	t[61]=8.86<br>p < 0.0001<br>Z[61]=900.5<br>p < 0001
1082 ± 158ms	-	1253.5 ± 211ms<br>*+15.8%*	t[61]=6.17<br>p < 0.0001	1157 ± 172ms	-	1347 ± 223ms<br>*+16.4%*	t[61]=6.37<br>p < 0.0001
-	1247 ± 236ms	1253.5 ± 211ms<br>*+0.5%*	t[61]=0.20<br>p = 0.8450	-	1354 ± 267ms	1347 ± 223ms<br>*-0.5%*	t[61]=0.17<br>p = 0.8684

Picture-Word recognition:

Response time: **Trimmed means**	Response time: **Means**

Related	Unrelated	statistic	Related	Unrelated	statistic
2458 ± 562 ms	3323 ± 640 ms<br>*+35.2%*	t[59]=17.85 $p < 0.0001$	2539 ± 517 ms	3345 ± 598 ms<br>*+31.7%*	t[59]=19.11 $p < 0.0001$
